# Effects of Nurse Staffing, Work Environment, Education on Adverse Events in Nursing Homes

**DOI:** 10.1093/geroni/igaa057.276

**Published:** 2020-12-16

**Authors:** Seonhwa Choi, Eunhee Cho, Eunkyo Kim, Kyoungeun Lee, Soo Jung Chang

**Affiliations:** 1 Yonsei University, Seoul, Republic of Korea; 2 Tongmyong University, Pusan, Republic of Korea; 3 Gangneung-Wonju National University, Wonju, Republic of Korea

## Abstract

This study examined the effect of registered nurse (RN) staffing level, work environment, and education on adverse events experienced by residents in nursing homes. A cross-sectional study was conducted with 216 RNs working in nursing homes who were selected using random stratified sampling by location and bed size. Self-reported questionnaires regarding staffing level, work environment, education level, adverse events, and nurse characteristics were administered. Data from the National Health Insurance Service were used to describe nursing home characteristics. Both multiple and multinomial logistic regressions were used to control for the characteristics of nurses and nursing homes, and investigate the effects of nursing staffing level (number of older adults assigned to a nurse), work environment (Practice Environment Scale of the Nursing Working Index), and level of nursing education on the adverse events experienced by residents. An increase of one resident per RN was significantly associated with a higher incidence of pressure ulcers (OR= 1.019, 95% CI=1.004-1.035). Poor work environment increased the incidence of adverse events such as pressure ulcers (OR= 3.732, 95% CI=1.155-12.056) and sepsis (OR=3.871, 95%CI=1.086-13.800). Compared to RNs with a baccalaureate or higher, RNs with diplomas reported increased incidence rates of pressure ulcers (OR=2.772, 95%CI= 1.173-6.549). RN staffing, work environment, and education level affect the incidence of pressure ulcers, and the work environment affects the incidence of sepsis among residents in nursing homes. Policy-wise, improving the level of nurse staffing, nursing work environment, and nursing education will improve health outcomes of residents.

